# Exploring the Multifunctional Roles of Betaine: Traditional Applications, Emerging Technologies, and Green Chemistry Innovations

**DOI:** 10.3390/foods15040737

**Published:** 2026-02-16

**Authors:** Yinuo Liu, Qiuxiao Li, Ruijia Liu, Zelong Wang, Shuna Zhao

**Affiliations:** 1 School of Food and Health, Beijing Technology and Business University, Beijing 100048, China; liunoe0408@163.com (Y.L.);; 2 Key Laboratory of Geriatric Nutrition and Health, Ministry of Education, Beijing Technology and Business University, Beijing 100048, China

**Keywords:** betaine, traditional applications, cocrystals, deep eutectic solvents (DESs), green chemistry, bibliometric analysis

## Abstract

Betaine, a simple natural zwitterion, is currently attracting widespread attention. Although historically labeled as an osmoregulator in agriculture and a methyl donor in animal nutrition, the molecule is now being repositioned at the forefront of green chemistry and materials science due to its unique physicochemical structure. This review critically explores the expanding horizon of betaine applications, bridging the gap between its established biological functions and its emerging roles in recently reported technologies, such as deep eutectic solvents (DESs), cocrystal engineering, and sustainable polymer synthesis. Beyond summarizing its versatile functionality across biomedicine, food science, and industrial formulations, we provide a comprehensive bibliometric analysis to map the evolution of research trends, identifying a clear focus toward industrial ecology and advanced materials. By synthesizing current advancements and discussing potential future directions, this work highlights betaine not merely as a supplement, but as a versatile molecular component with potential applications in sustainable materials and chemical engineering processes.

## 1. Introduction

Betaine, a natural quaternary ammonium compound, has emerged as a primary research subject due to its diverse properties, including osmoregulation, methyl transfer capabilities, participation in hydrogen bond networks, and potential for supramolecular assembly. Characterized by a permanent zwitterionic nature, betaine serves as the polar headgroup motif for amphiphilic derivatives; however, lacking a hydrophobic tail, the molecule itself acts as a highly water-soluble osmolyte rather than a surfactant. This unique physicochemical versatility and biocompatibility, support its widespread application across agriculture, biomedicine, chemical engineering, and food science. In recent years, advancements in crystal engineering, green chemistry, and materials science have further expanded the theoretical and practical scope of betaine research, establishing it as a versatile molecular platform supporting emerging technologies such as cocrystals and deep eutectic solvents (DESs).

Despite the extensive literature discussing the traditional functions of betaine, a comprehensive review integrating classical applications with recent breakthroughs in supramolecular assembly and solvent engineering is lacking. To address this, we present a narrative review of the primary applications of betaine, analyze the current research landscape and trends via bibliometric analysis, and highlight novel scientific advancements. Furthermore, we integrate emerging research directions to emphasize the role of betaine in “sustainable molecular design”.

**Figure 1:**
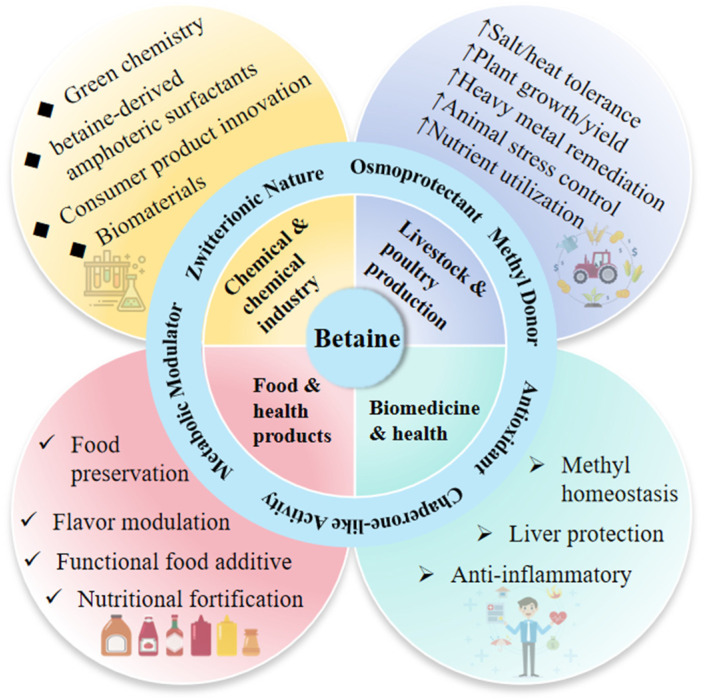
Four traditional fields of Betaine.

The primary domains of betaine’s traditional applications are summarized in Figure 1. Functioning as a potent osmoprotectant and metabolic modulator, betaine not only significantly enhances stress resistance and performance in livestock and poultry farming but also demonstrates superior hepatoprotective and anti-inflammatory activities in biomedicine. Moreover, its functional attributes extend to food preservation and serving as a structural precursor for surfactant synthesis, demonstrating its high value in diverse applications.

## 2. Main Applications of Betaine

### 2.1. Applications in Animal Nutrition and Husbandry

The efficacy of betaine as a methyl donor and osmolyte is characterized by notable variability across species, dosages, and environmental contexts (Table 1). While recognized as a functional feed additive, its performance is not universally consistent, necessitating a critical evaluation of its metabolic impact and practical limitations.

Betaine interacts with cellular homeostasis through antioxidant capacity and osmotic regulation. In swine, supplementation has been reported to improve pork quality by modulating initial pH and lipid stability [1]. However, the magnitude of these effects often depends on basal diet composition and pre-slaughter stress levels, indicating inconsistency in product quality outcomes. In poultry, the response to betaine is highly context-dependent, with benefits primarily observed under heat stress. While specific studies report improvements in growth performance and feed conversion ratios (FCR) under high temperatures [2,3], these results are often not replicated in thermoneutral environments, suggesting betaine functions more as a stress-mitigating agent than a primary growth promoter. Furthermore, the partial replacement of methionine with betaine in laying hens presents a metabolic trade-off [4]; the optimal replacement ratio remains a subject of debate, as over-supplementation risks disrupting one-carbon metabolism.

The impact on ruminants and aquatic species further illustrates species-specific variability. Meta-analyses indicate increases in milk yield for dairy cows and weight gain for beef cattle [5], yet the rumen bypass efficiency of unprotected betaine remains a technical bottleneck. Similarly, in aquaculture, growth-promoting effects appear restricted to carnivorous and omnivorous species [6]. These findings suggest that the metabolic utilization of betaine is governed by specific digestive physiology rather than a uniform biological mechanism.

Regulatory standards, such as those from the European Food Safety Authority (EFSA), provide a safety baseline of 2000 mg/kg [7]. However, these standards are frequently extrapolated from porcine models, representing a knowledge gap in long-term safety data for diverse livestock species. Future research must address the underlying causes of these inconsistent responses and the metabolic risks of concurrent administration via multiple routes.

**Table 1:** Betaine characteristics in livestock and poultry productions field and related research summary.

Mechanism	Model/Example	Dose & Method	Key Outcome	Ref.
Improvement of meat quality & oxidative stability	Pigs (RYR1 gene mutation, heterozygous Nn)	Betaine (1.25 g/kg feed) or Creatine (2.0 g/kg feed); 30 days pre-slaughter	Vs. Control (Nn): Betaine: Initial pH ↑, Drip loss ↓; Both: Oxidative stability ↑	[1]
Osmoprotection & antioxidant	Broiler chicken (heat stress)	0.10% *w*/*w* betaine in feed	Weight gain +84 g; FCR improvement 4.6 pts	[2]
Enhances growth performance, meat quality, and antioxidant capacity	Broiler chickens	Anhydrous betaine (500 or 1000 mg/kg) or hydrochloride betaine (642.23 or 1284.46 mg/kg)	↓ Drip loss and lipid peroxidation; ↑ growth performance, muscle yield, and antioxidant status	[3]
Methyl donor substitution & immunomodulation	Laying hens (Bovans brown)	Dietary replacement of choline with betaine (0, 25, 50, 100%) for 12 weeks	Egg weight & Egg mass ↑; Yolk color intensity ↑; Newcastle disease (ND) antibody titer ↑; Serum lipids & liver enzymes: No significant change	[4]
Methyl donor & osmoregulation	Ruminants (meta-analysis)	10–15 g betaine/day in diet	Milk yield +1.0 kg/d; ADG +0.019 kg/d	[5]
Enhanced nutrient utilization	Aquaculture (meta-analysis)	0.99% *w*/*w* betaine in feed	Specific growth rate & FCR ↑	[6]
Antioxidant defense & mitigation of oxidative stress	Rats (Cadmium-induced testicular toxicity)	1.5% (*w*/*w*) of total diet (oral) for 10 days	Testicular TBARS ↓; CAT & SOD activities ↑; pathological changes prevented	[8]
Enhances glucose uptake, insulin sensitivity, protein utilization	Ruminants	Betaine-based feed additive (betaine + biotin + chromium) at 3 or 6 g/kg DM	Improved nutrient utilization without affecting rumen fermentation or nitrogen balance	[9]

Notes: ↑ indicates an increase or improvement; ↓ indicates a decrease or reduction.

### 2.2. Biomedicine and Health

As a natural bioactive compound, betaine has been extensively studied in biomedicine [10], exhibiting biological activities across various pathological contexts (Table 2). However, current evidence is derived primarily from cellular and animal models, providing insights into betaine-mediated metabolic regulation and cytoprotective effects rather than established clinical efficacy.

#### 2.2.1. Alleviation of Liver Injury

Betaine impacts hepatoprotection primarily through the regulation of sulfur amino acid metabolism. While animal models of ethanol-induced injury show that betaine can restore depleted levels of S-adenosylmethionine (SAM) and glutathione [11], the clinical translation of these findings remains inconsistent. The restorative effect on antioxidant defenses is often observed in acute or controlled nutritional interventions, yet its efficacy in chronic human liver pathologies is less validated. Regarding non-alcoholic fatty liver disease (NAFLD), maternal supplementation has been reported to influence offspring liver health via gut microbiota modulation [12]. Mechanistically, betaine acts as a methyl donor for betaine-homocysteine methyltransferase (BHMT), promoting the remethylation of homocysteine (HCY) to reduce plasma levels, thereby driving the methionine cycle—a critical mechanism for alleviating NAFLD.

Mechanistically, betaine is reported to inhibit steatosis and apoptosis in NAFLD by modulating autophagy and the AMP-activated protein kinase (AMPK) signaling pathway [13]. While induction of autophagy appears to be an upstream event for mitigating endoplasmic reticulum stress, the precise crosstalk between these pathways remains partially characterized [14]. Recent research reveals that betaine modulates autophagy-related pathways. Betaine has been reported to enhance ATG3 stability through the SAM/m6A/YTHDF1 axis, which is associated with reduced stem-like characteristics in experimental models of hepatocellular carcinoma [15]. Experimental evidence also indicates that betaine affects hepatic antioxidant defenses and mitochondrial function, both of which are closely related to cellular stress responses and hepatocyte integrity [16].

In summary, current studies indicate that betaine is involved in several hepatoprotective mechanisms. These include regulation of sulfur amino acid metabolism, modulation of autophagy–AMPK signaling, attenuation of oxidative stress, and maintenance of mitochondrial function. Most evidence comes from cellular and animal models. These findings provide mechanistic insights into liver protection under experimental conditions. Although existing studies provide solid preclinical evidence, further exploration is needed to elucidate the precise molecular mechanisms and validate its value in human liver diseases, ultimately establishing a basis for its clinical application as a targeted hepatoprotective strategy.

#### 2.2.2. Prevention of Cardiovascular Disease

Similar to its role in alleviating liver injury, betaine participates in homocysteine metabolism by serving as a methyl donor, thereby significantly reducing plasma total homocysteine (tHCY) levels. This regulatory pathway represents a fundamental mechanism for cardiovascular disease prevention [17]. While meta-analyses indicate that daily supplementation reduces plasma HCY levels in healthy adults [18], the clinical significance of this reduction for cardiovascular endpoints remains debated. In specific populations such as Chinese adults with hyperhomocysteinemia, betaine contributes to remethylation in a non-folate fortified context [19]. Furthermore, supplementation in physically active men suggests metabolic safety in normolipidemic populations [20].

However, the observed decrease in tHcy does not always translate into a proportional reduction in cardiovascular events, suggesting that Hcy may function as a biomarker rather than a direct causative factor. Beyond metabolic regulation, betaine has been reported to mitigate toxin-induced cardiotoxicity in animal models by attenuating oxidative stress and inflammatory responses [21]. The relevance of these acute rodent studies to chronic, low-level human exposure is unverified.

#### 2.2.3. Regulation of Systemic Metabolism

Betaine is closely linked to the fundamental physiological and metabolic homeostasis of the host. Experimental studies indicate that betaine supplementation can ameliorate high-fat diet (HFD)-induced obesity and metabolic syndrome in mice by modulating gut microbiota composition—specifically increasing the abundance of *Firmicutes*, *Lactobacillus*, and *Bifidobacterium*. This modulation is associated with enhanced short-chain fatty acid (SCFA) production and the regulation of host gene expression via the microbiota-dependent miR-378a/YY1 axis, thereby contributing to improved glucose homeostasis and adipose tissue function [22]. Furthermore, maternal betaine supplementation during lactation has been shown to increase betaine concentrations in breast milk, which is associated with reduced adiposity and improved glucose homeostasis in the offspring. These effects are mediated in part by the early-life enrichment of *Akkermansia muciniphila* and the long-term enhancement of intestinal goblet cell numbers, suggesting a potential link between milk-derived betaine and metabolic health programming [23].

Epidemiological evidence further supports the metabolic relevance of betaine. In women with dichorionic twin pregnancies, higher second-trimester plasma betaine levels were significantly associated with a lower risk of gestational diabetes mellitus (GDM), suggesting a protective role in glucose metabolism during high-risk pregnancies [24]. Similarly, a case–control study revealed that lower serum betaine levels and a reduced betaine/choline ratio were significantly associated with increased GDM risk and impaired glucose tolerance, indicating that betaine contributes to glycemic stability and may serve as a metabolic biomarker for GDM [25].

At the cellular levels, betaine protects mouse Leydig cells under hyperglycemic conditions by regulating steroidogenic gene expression, alleviating endoplasmic reticulum (ER) stress, and activating the Nrf2-mediated antioxidant pathway, thereby restoring testosterone production and improving cell viability [26]. In mouse models, betaine supplementation appears to influence glucose uptake and glycogen metabolism, potentially through the Notch signaling pathway [14]. Moreover, betaine supplementation has been shown to influence hepatic insulin signaling in high-fat diet (HFD)–induced obese mice. Experimental studies report partial restoration of hepatic and serum betaine levels, along with enhanced tyrosine phosphorylation of insulin receptor substrate-1 (IRS1) and activation of protein kinase B (Akt). In these models, betaine treatment is also associated with increased hepatic glycogen content and reduced indicators of liver injury. In vitro, betaine reverses insulin resistance in primary human hepatocytes by enhancing insulin signaling pathways [27].

These findings suggest that betaine interacts with the gut microbiota-host metabolic axis to influence insulin sensitivity and glucolipid metabolism across the liver, digestive tract, and gonads. While these pathways indicate a potential role for betaine in managing metabolic syndrome and type 2 diabetes mellitus (T2DM), current evidence relies heavily on specific physiological contexts. The proposed efficacy in mitigating metabolic complications remains contingent upon reconciling inconsistent results between clinical observations and animal models. Furthermore, the long-term impact of betaine on systemic endocrine balance across different target organs is not fully characterized, necessitating a more cautious interpretation of its role as a standardized nutritional intervention.

#### 2.2.4. Amelioration of Muscle Atrophy

Current research on muscle atrophy relies on disparate experimental models. In C2C12 myotubes, betaine directly attenuates TNF-α-induced atrophy by sustaining *Myh1* expression [28]. Conversely, studies in aged mice link betaine to systemic metabolic regulation. It elevates SAM levels to modulate the Samtor–mTORC1 axis for protein synthesis [29]. Independent evidence in rodent muscle suggests betaine also promotes autophagic flux via the Mettl21c/p97/VCP pathway [30]. While in vitro models isolate direct cytoprotection, whereas in vivo efficacy depends on systemic methionine cycling. Furthermore, the simultaneous support of mTORC1-driven anabolism and autophagic flux represents a biological paradox, as these processes are typically antagonistic. Future work must resolve whether these pathways are spatially separated or temporally regulated within skeletal muscle.

#### 2.2.5. Anti-Inflammatory and Immunomodulatory Properties

Betaine exhibits anti-inflammatory activity across various tissues, primarily targeting NF-κB and NLRP3 signaling. In murine neuroinflammation, it suppresses the TLR4/NF-κB pathway [31]. In BV2 microglial cells, betaine specifically inhibits amyloid-β-induced NLRP3 activation [35]. Studies on depression models show it promotes a microglial phenotype shift from M1 to M2 [36]. Beyond the nervous system, betaine reduces retinal inflammation in autoimmune uveitis by downregulating VCAM-1 [34]. In hepatic models, it mitigates inflammation by suppressing the NLRP3/ASC/Caspase-1 axis [32]. Vascular protection is also observed in ApoE−/− mice, where betaine reduces atherosclerosis via NF-κB inhibition [33]. Despite these findings, immune sensitivity varies significantly between acute chemical induction and chronic genetic models. A major limitation is the widespread reliance on high-dose protocols that exceed standard dietary intake. Future research must shift focus to long-term, low-dose exposure to determine if these immunomodulatory benefits translate to human chronic diseases.

#### 2.2.6. Other Biological Activities

Beyond its clinical applications, betaine demonstrates significant value in sports nutrition and stress resilience. Betaine supplementation (2 g/day for 14 weeks) enhanced aerobic capacity (VO_2_max), muscular strength (1-RM), and repeated sprint ability in young professional soccer players, suggesting it is a beneficial nutritional strategy for improving athletic performance during competitive seasons [37]. In neuroprotection studies, betaine pretreatment mitigates ZnO nanoparticle-induced depressive-like behaviors, oxidative stress, and hippocampal damage in mice. It reduces immobility time in behavioral tests, restores antioxidant enzyme activity (SOD, GPx), and improves hippocampal histopathology [38]. Similarly, betaine alleviates CFA-induced chronic pain-related depressive-like behavior by inhibiting glial activation and promoting a phenotypic shift in microglia (M1 → M2) and astrocytes (A1 → A2), thereby reducing hippocampal cytokines (IL-1β, IL-6, IL-18) while enhancing IL-10 levels [39].

Betaine has also been investigated for its role in modulating gut-associated physiological responses under stress and inflammatory conditions. Experimental studies in animal models suggest that betaine intake can influence gut microbiota composition, short-chain fatty acid profiles, and systemic inflammatory markers, which are key factors linking diet to intestinal and metabolic health [40]. In models of acute liver injury, betaine has been reported to alleviate intestinal barrier dysfunction by regulating TLR4/MyD88 signaling, enhancing tight junction protein expression, and reshaping gut microbial communities [41]. Similarly, in chemically induced colitis models, betaine administration has been associated with improved intestinal barrier integrity, reduced oxidative stress, and modulation of inflammasome-related pathways [42].

From a food science and nutrition perspective, these findings highlight the potential relevance of betaine as a diet-derived component influencing gut barrier function and host–microbiota interactions, although current evidence is largely derived from experimental models and requires further validation in food-based and human dietary contexts.

#### 2.2.7. Construction of Functional Polymers

Amino-acid-derived polyzwitterions and polybetaines (PBs) represent emerging polymer classes with significant potential in biomedical applications, such as enhancing tumor permeability and improving bio-interface performance. The quaternization of amino-acid-derived polyzwitterions combines the advantages of both PBs and traditional polyzwitterions, yielding polymers with broader pH stability, maintained chirality, and excellent mammalian cell tolerance (≤1 mg mL^−1^). These materials utilize the inherent zwitterionic nature of betaine moieties to achieve low fouling properties and target specificity, making them promising candidates for advanced biological applications [43].

**Table 3:** Betaine characteristics in chemical and chemical industry field and related research summary.

Mechanism	Model/Example	Dose & Method	Key Outcome	Ref.
Synergistic effects of SCG and CAPB in complex system; improved performance compared to SCG alone	SCG/CAPB System	Visible-ultraviolet spectrophotometry, surface tension meter, POM, SAXS	SCG and CAPB mixture enhances phase behavior and performance in personal care formulations	[44]
Mild surfactancy; low skin irritation	Mild personal-care formulation	60 μL applied via 24 h occlusive patch test (24 volunteers); standardized cleaning assay (model dirt)	Reduced irritation; good cleansing efficacy	[45]
Modulation of surface activity & micellar self-assembly via alkyl chain length	CAPB components (Pure amidopropyl betaines with varying tail lengths)	Physicochemical analysis (SANS, Tensiometry, Foaming studies, Microscopy)	Increased alkyl tail length correlates with higher surface activity; Altered micelle geometries; Modulated adsorption dynamics & foaming; Enables optimization of surfactant properties via feedstock selection	[46]
Safety assessment & Toxicological evaluation (Irritation, Sensitization, Systemic toxicity)	Cosmetic formulations (Leave-on/Rinse-off) & Rats (92-day repeated dose toxicity)	Usage limits: 30% (rinse-off), 6% (leave-on); NOAEL: 250 mg/kg/day (Oral)	Mild skin/eye irritation & sensitization observed; Systemic exposure 0.0012–0.93 mg/kg/day; Margin of Safety (MOS) > 100; Concluded safe under current usage conditions	[47]

### 2.3. Applications in Consumer Products

Betaine is widely used in personal care and industrial products. It plays two main roles: functioning directly as a moisturizer, and serving as a key precursor for zwitterionic surfactants like cocamidopropyl betaine (CAPB). Specific applications are detailed in Table 3. A critical distinction exists in their application controllability: natural betaine provides predictable hydration benefits, whereas the performance of CAPB is highly variable and dependent on synthesis conditions. Specifically, the surface activity and foaming stability of CAPB are dictated by the alkyl chain length distribution [48]. Consequently, consistent surfactant performance requires precise control over feedstock composition, as minor variations in chain length can significantly alter rheological behavior. Toxicological evaluations indicate a Margin of Safety (MOS) exceeding 100 for CAPB within regulated limits (≤30% for rinse-off; ≤6% for leave-on products) [46,47]. However, clinical safety is not intrinsic to the surfactant alone but relies on minimizing synthesis byproducts, such as amidoamines, which are potential sensitizers.

In complex surfactant systems, CAPB functions as a co-surfactant to modulate phase behavior. Evidence indicates that it broadens solubility regions in sodium cocoyl glycinate formulations [44]. Furthermore, it mitigates the harshness of sodium lauryl sulfate systems while maintaining cleaning efficacy [45]. Crucially, these synergistic benefits are not universal; the capacity of CAPB to optimize phase stability and reduce irritation is strictly conditional, relying on precise adjustments to pH and ionic strength.

### 2.4. Applications in Food Science

Betaine has attracted significant attention in the food industry, as a multifunctional ingredient in functional foods and nutraceuticals. Its primary applications, summarized in Table 4, include functional additives, preservatives, flavor modulators, and nutritional fortifiers. Furthermore, this subsection discusses the safe inclusion limits and regulatory standards associated with betaine, ensuring compliance and consumer safety in its commercial application.

#### 2.4.1. Functional Food Additive

Betaine is utilized in food matrices primarily for its chemical stability, though its functional impact varies by application method. In bakery systems, the timing of incorporation is critical. Adding betaine prior to dough mixing has been shown to impair gluten network development, resulting in reduced gas retention and increased crumb hardness. In contrast, late-stage addition minimizes interference with protein structure, thereby improving loaf texture [49].

Beyond direct addition, betaine-derived structures facilitate the processing of lipid-based ingredients. For instance, the amphoteric surfactant CAPB modifies phase behavior during the enzymatic synthesis of monoacylglycerides (MAGs). This application enhances catalytic selectivity, serving as a process aid for developing emulsifiers used in food and cosmetic systems [50]. Regarding natural sources, beetroot powder is explored as a whole-food alternative to purified betaine. Substituting flour with beetroot powder in biscuits increases mineral content and lowers glycemic responses, although the sensory acceptance relies on optimizing substitution ratios to balance flavor profiles [51]. Toxicological assessments following OECD guidelines indicate no genotoxic effects for betaine under standard conditions, supporting its safety profile for food applications [52].

#### 2.4.2. Food Preservation & Quality Maintenance

Exogenous betaine application is investigated as a strategy to mitigate physiological stress in postharvest horticulture. In cold-stored pomegranates, immersion in 20 mM betaine was reported to alleviate chilling injury (CI) by maintaining membrane integrity and preserving ascorbic acid levels [53]. A comprehensive review attributes these anti-CI effects to the regulation of oxidative metabolism and the expression of cold-resistance genes [54].

Similarly, in blueberries, treatment with 10 mM betaine was associated with delayed senescence and enhanced fruit firmness. These outcomes are linked to the upregulation of enzymes involved in energy metabolism and antioxidant defense [55]. Broader physiological assessments suggest that betaine modulates ethylene production and cell wall degradation, thereby reducing storage-related deterioration [56]. Extending to pre-harvest cultivation, foliar spraying of betaine has been shown to improve soluble solid content and polyphenol levels in sweet cherries [57]. Despite these benefits, efficacy appears to be species-specific and dependent on application methods (e.g., dipping versus spraying). Furthermore, widespread industrial adoption requires evaluating cost-effectiveness compared to conventional synthetic preservatives.

#### 2.4.3. Flavor Modulation

Betaine modulates flavor profiles in both animal nutrition and food formulation. In aquaculture, it acts as a feeding stimulant for sole (*Solea solea*), enhancing food-seeking behavior [58]. Behavioral assays with juvenile grouper further indicate that betaine synergizes with amino acid mixtures to increase feed preference [60]. Regarding human food systems, betaine serves as a flavor precursor in plant-based seafood analogues. Thermal processing facilitates its conversion into volatile compounds, such as pyrazines and thiazoles, creating crustacean-like aromas [59]. In ruminant nutrition, rumen-protected betaine influences meat quality. Supplementation in growing lambs alters fatty acid profiles and increases flavor-related amino acids, thereby modifying post-slaughter flavor characteristics [61]. A critical distinction exists across these applications: betaine functions via chemoreception in aquatic species, whereas in food processing, it undergoes chemical degradation (Maillard reaction). Furthermore, despite its functional benefits, the high cost of purified betaine relative to synthetic flavor enhancers remains a primary constraint for widespread industrial adoption.

#### 2.4.4. Regulatory Requirements and Application Limits

Global regulatory frameworks governing betaine products vary by region, reflecting distinct safety assessments and usage conditions, as outlined in Table 5.

Europe, particularly the European Union (EU), is a primary production hub for sugar beets and leads natural betaine research and application. Following EU food safety evaluations, betaine was classified as a “Novel Food” in 2017, with a recommended daily intake of 400 mg. By 2019, it was further recognized as a functional ingredient in sports nutrition products under the expanded scope of novel food applications. In 2019, the European Union formally authorized betaine as a novel food for market use, specifying permitted food categories, maximum inclusion levels, labeling requirements, and provisions for data protection. Notably, the proprietary data protection for this authorization was valid until 22 August 2024. Comprehensive specifications regarding the product description, composition, heavy metal limits, and microbiological criteria for this novel food are provided in Table 6.

### 2.5. Limitations of Betaine Application

Industrial application of betaine faces several specific constraints. Economic viability is limited by purification costs; extraction from sugar beet molasses is energy-intensive and depends on raw material availability, affecting price stability compared to petroleum-based alternatives. From a sustainability perspective, the environmental footprint of downstream chemical modifications requires verification. Claims of betaine being a sustainable building block currently lack comprehensive life cycle assessments to account for the energy and solvents used in these processes. Furthermore, scalability is restricted by global regulatory discrepancies regarding dosages and health claims, alongside a lack of standardized long-term safety data. These techno-economic and regulatory hurdles indicate that the practical implementation of betaine-based platforms depends on addressing specific industrial bottlenecks rather than relying on its bio-based origin.

## 3. Emerging Frontiers in Betaine Research: Cocrystals and Deep Eutectic Systems

While the traditional applications of betaine are well-established, its inherent limitations—such as complex dosage regulation in biomedical contexts and performance sensitivity in industrial formulations—have prompted researchers to explore innovative strategies to enhance its functionality and scope. Consequently, engineering betaine via multi-component systems, specifically cocrystals and DESs, has emerged as a promising approach. Although much of this research originates from pharmaceutical and materials science fields, the underlying principles are increasingly being explored for their relevance to food ingredient stabilization, delivery, and processing systems.

A literature search was conducted in the Web of Science (WOS) database using “betaine deep eutectic solvent” and “betaine cocrystal” as topic keywords. The data source was limited to the Web of Science Core Collection, with the publication period restricted from 1 January 2000, to 20 October 2025, and the language refined to English. After deduplication using CiteSpace 6.3.R1, a total of 608 English-language publications were obtained. The Web of Science (WOS) database was used for literature data collection. Each retrieved record was saved in RefWorks format and as plain text files containing full records and cited references. The downloaded data were then processed using CiteSpace 6.3.R1 for deduplication and format conversion. Finally, the resulting TXT-format files were used to generate relevant visualization maps for bibliometric analysis.

### 3.1. Research Landscape

**Figure 2:**
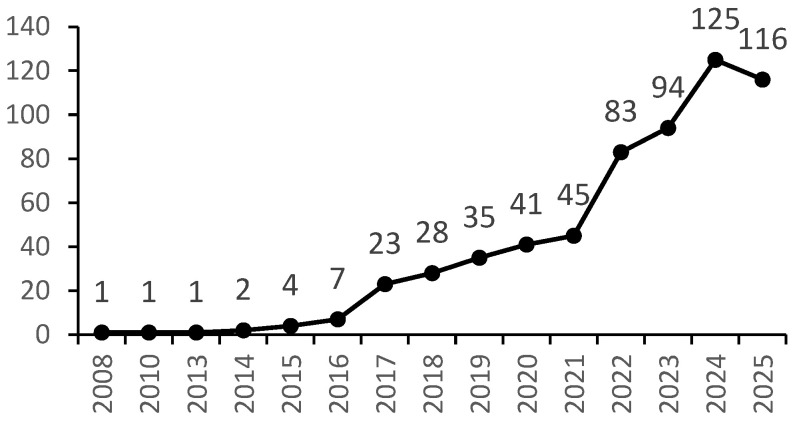
Publication trends in betaine research on cocrystals and deep eutectic solvents.

The scientific community’s growing focus on betaine-based multicomponent systems is clearly reflected in publication trends. Bibliometric analysis reveals a significant surge in publications concerning betaine research in the fields of cocrystals and DESs, with a notable acceleration starting in 2022 (Figure 2). This trend suggests a shift from investigating betaine solely as a bioactive compound toward its use as a functional molecular component in structured systems, including those with potential relevance to food and nutraceutical applications.

**Figure 3:**
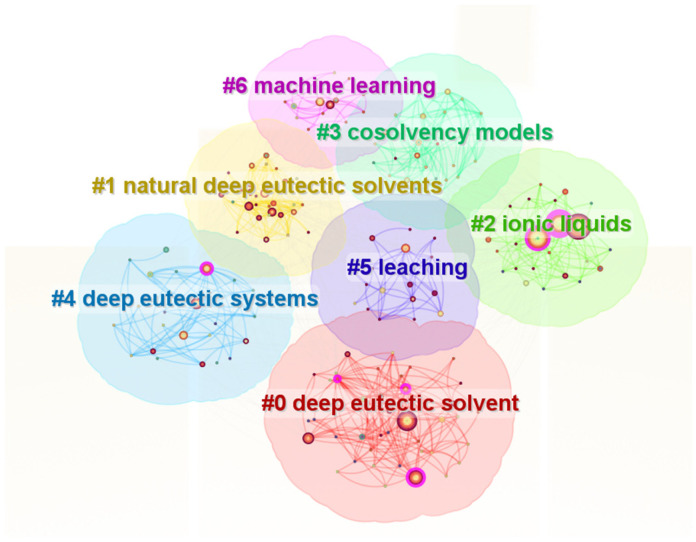
Keyword clustering map of betaine research in cocrystals and deep eutectic solvents.

The keyword cluster map (Figure 3) further illustrates a tightly interconnected and dynamic research landscape. The central theme of “Deep Eutectic Solvents” is closely associated to “Natural Deep Eutectic Solvents” (NADES) and the related domain of “Ionic Liquids”, positioning betaine-based solvent research within the broader framework of sustainable alternative media. Notably, clusters related to extraction, leaching, and solvent design indicate growing interest in applying these systems to the recovery and stabilization of food-derived bioactive compounds, while data-driven approaches such as machine learning highlight advances toward rational formulation design.

From an interpretative perspective, the rapid expansion of DES- and cocrystal-related keywords suggests a paradigm shift: research is moving beyond investigating betaine solely as a functional additive toward utilizing it as a structural building block in multicomponent engineering. While traditional applications appear comparatively mature, the strong concentration of keywords in pharmaceutical and materials science domains indicates a disciplinary bias. This disparity underscores a significant translational gap, suggesting that the advanced supramolecular strategies currently dominating non-food fields hold untapped potential for addressing stability and delivery challenges within food systems. Collectively, this bibliometric evidence indicates that research on betaine-based cocrystals and DESs is expanding and increasingly intersecting with challenges relevant to food science, green processing, and ingredient functionality.

### 3.2. Mechanisms of Cocrystal and Deep Eutectic Formation

The formation of both cocrystals and DESs is governed by supramolecular interactions, particularly hydrogen bonding. Betaine is well suited for these systems due to its zwitterionic structure, which combines a strong hydrogen bond-accepting carboxylate group with a quaternary ammonium moiety.

**Figure 4:**
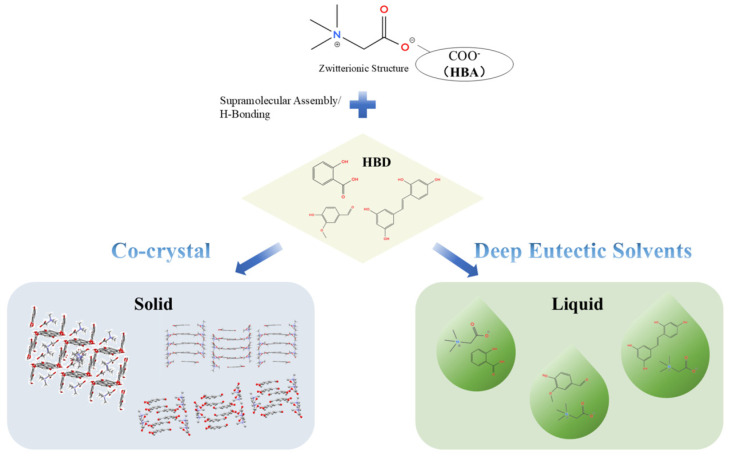
Figure of formation mechanism of betaine cocrystal and deep eutectic.

The formation mechanisms of betaine cocrystals and deep eutectic systems are illustrated in Figure 4. In cocrystal systems, betaine can act as a coformer by establishing specific hydrogen-bonding interactions with partner molecules at defined stoichiometric ratios. While such systems have been extensively studied in pharmaceutical contexts, similar interaction principles may be applicable to the stabilization and delivery of food bioactives, flavors, and nutraceutical ingredients. In DESs, betaine typically functions as a hydrogen bond acceptor. When combined with suitable hydrogen bond donors, such as polyols or organic acids, extensive hydrogen-bonding networks form, leading to a significant depression of the melting point and the formation of liquid solvents at ambient conditions. These properties provide a physicochemical basis for developing alternative solvent systems for food processing, extraction, and enzyme stabilization.

## 4. Applications of Betaine in Cocrystal and Deep Eutectic Systems

### 4.1. Biomedical Applications

Cocrystallization involving betaine has been widely explored to improve the solubility and stability of poorly water-soluble compounds. Although many reported examples involve pharmaceutical active ingredients, the underlying strategy offers conceptual guidance for improving the functional performance of bioactive compounds relevant to food and nutrition. Studies on flavonoids such as baicalin, rutin, and quercetin demonstrate enhanced solubility and dissolution behavior when incorporated into betaine-based cocrystal systems [64,65,66]. Similarly, resveratrol–betaine cocrystals show improved aqueous solubility and absorption characteristics [67]. features that are also desirable for food-derived polyphenols and nutraceutical formulations.

In DES research, betaine-based NADESs have been extensively investigated as alternative solvent systems. From a food science perspective, particular attention has been given to enzyme stabilization and protein protection under processing-relevant conditions. For example, a trehalose–betaine NADES exhibits strong stabilizing effects on trypsin structure and activity under thermal stress [68]. Betaine–glycerol systems have also been shown to enhance the thermal stability of microbial transglutaminase, an enzyme widely used in food processing [69]. Furthermore, molecular dynamics simulations indicate that betaine–sorbitol NADESs can protect β-lactoglobulin against thermal denaturation by strengthening internal hydrogen bonding and reducing solvent exposure, suggesting potential applicability in high-temperature food processing environments [70].

In summary, betaine-based cocrystals and deep eutectic systems provide versatile platforms for modulating solubility, stability, and functional performance of bioactive compounds and proteins. While many studies originate from pharmaceutical or materials science research, the concepts and mechanisms described offer transferable insights for food formulation, ingredient delivery, and bioprocessing applications, provided that system-specific validation is conducted within food matrices.

### 4.2. Applications in Cosmetics

Betaine cocrystallization is investigated as a strategy in cosmetic formulations, particularly for optimizing the physicochemical properties of poorly soluble or irritant compounds such as salicylic acid. Experimental data suggest that betaine-salicylic acid cocrystals can mitigate cytotoxicity and skin irritation while retaining anti-inflammatory and antioxidant activities [71]. Regarding nanocarrier construction, the betaine-glycerol DES has been identified as an effective hydrophilic matrix for preparing high-performance, surfactant-free nanoemulsions. Through co-assembly with hydrophobic DESs, this system facilitates the encapsulation and solubilization of volatile essential oils, thereby improving storage stability and bioactivity of the formulation, offering a new strategy for green nanocarriers in the cosmetic and pharmaceutical sectors [72]. For raw material extraction, a betaine-sucrose (2:1 molar ratio) DES system replaced organic solvents for the extraction of essential oils from tea tree and lemon grass. This process enriched active terpene compounds under mild conditions, increasing yields by 1.7–2.5 fold [73]. In anti-aging applications, a new betaine-succinic acid-based DES system was developed to enhance the bioavailability of elastin peptides. This solvent acts as a carrier to facilitate the transdermal permeation and appears to stimulate the secretion of procollagen I and elastin. Studies report a combined effect with the payload in improving skin elasticity, inhibiting melanin deposition, and resisting photoaging [74]. Additionally, computational modeling indicates stable binding interactions between silymarin and DES solvents. This system modulates skin permeability and collagen synthesis, suggesting utility in anti-aging cosmetics [75].

In summary, the application of betaine in the cosmetic sector has evolved beyond its traditional role as a humectant. Through cocrystallization and deep eutectic technologies, it functions in raw material modification, solvent substitution, and delivery system construction. While these applications offer benefits regarding irritation reduction and transdermal absorption, the long-term stability of DES-based formulations in complex cosmetic matrices (e.g., interaction with emulsifiers or preservatives) remains a critical variable requiring further stability testing.

### 4.3. Green Chemistry and Sustainable Development

Betaine-based cocrystal materials are studied in green chemistry, particularly for their utility in developing stabilized preservatives and controlled-release systems. For instance, the thymol-betaine cocrystal acts as a stable hygroscopic complex that effectively inhibits the growth of *Aspergillus flavus* and significantly reduces contamination levels of aflatoxin B_1_ (AFB_1_) [76]. Due to its ability to modulate humidity and microbial stability, this technology presents an alternative for food preservation and the storage of agricultural commodities.

Regarding Deep Eutectic Solvents (DESs), betaine-based solvents serve as reaction media and functional reactants. Specifically, cinchona-squaramide organocatalysts immobilized within a betaine-sorbitol-water DES system achieved Michael addition reactions with high yields (up to 99%) at 1 mol% loading, maintaining activity over nine cycles [77]. In nanomaterial synthesis, a betaine-phenol DES facilitated the synthesis of highly crystalline, water-stable sub-100 nm hexagonal wurtzite zinc oxide nanoparticles at relatively low temperatures (approximately 80 °C) [78]. These nanoparticles were integrated into PVA films for UV shielding, illustrating the utility of betaine DESs in sustainable synthesis and functional material design. Furthermore, Type III DESs based on quaternary ammonium salts (including betaine) serve as platforms for synthesizing metal nanoparticles (Ag, Au, Cu, etc.). In these systems, the DES functions simultaneously as a solvent, reducing agent and stabilizer, enabling one-step green preparation of nanomaterials in an anhydrous environment [79]. For agricultural waste valorization, adhering to green chemistry principles, researchers developed a task-specific betaine-lactic acid DES for recovering high-value polyphenolic compounds from grape pomace. Combined with ultrasound-assisted technology, this system disrupted plant cell walls, increasing the extraction yield of hydroxycinnamic acids [80]. Similarly, a volatile organic compound (VOC)-free betaine-citric acid DES system was developed to assist in photocatalytic lignin depolymerization. This system optimized viscosity to promote the selective cleavage of β-O-4 bonds (achieving a degradation rate of 85.62%), supporting the potential of betaine-based solvents in biomass valorization [81].

In summary, betaine-based materials serve as versatile platforms in green chemistry. Applications range from reaction media with high atom economy to templates for nanosynthesis. However, a major challenge for industrial upscaling is the high viscosity of many betaine-based DESs, which can impede mass transfer. Furthermore, the economic feasibility of recycling these solvents compared to conventional volatile organic compounds requires comprehensive life-cycle assessment.

### 4.4. Extraction and Separation Processes

Betaine-based DESs are investigated as solvents for extracting bioactive components. For example, a betaine-sorbitol NADES system was utilized for the ultrasound-assisted extraction of chlorogenic acid and caffeine from Robusta green coffee beans, proposing a sustainable alternative to organic solvents [82]. Regarding drug solubility, a betaine/ascorbyl glucoside (Bet/AA-2G) eutectic solvent improved the solubility of silymarin by 60.9% compared to ethanol solutions [75].

In selective extraction studies, a betaine-urea (1:2 molar ratio) system, combined with ultrasound, enriched rosmarinic acid (1.934 mg/g) from comfrey. Notably, it restricted the extraction of the hepatotoxic component, lycopsamine, to 0.018 mg/g, indicating higher selectivity compared to methanol extraction [83]. As described in Section 4.2, the betaine-sucrose system is also applied to plant essential oils. Compared to water or ethanol, this system increased the yields of terpinolene from tea tree oil and neral from lemon grass oil by 2.5-fold and 1.9-fold, respectively [73]. Additionally, Vorobyova et al. characterized a betaine-based DES where conductivity varies with water content. Experimental results show that this system, aided by ultrasound, disrupts cell wall barriers in grape pomace, achieving total phenol yields up to 92 mg EGE/g [80].

In summary, betaine-based DESs offer tunable hydrophilicity and hydrogen bonding capacity for natural product extraction. While they enhance extraction rates and selectivity for specific compounds, a major operational limitation is their high viscosity at room temperature, which often requires auxiliary energy (e.g., heat or ultrasound) to ensure adequate mass transfer. Furthermore, separating the extracted solutes from the DES matrix remains a challenge, potentially increasing the energy footprint of the downstream purification process.

### 4.5. Material Synthesis and Processing

In materials science, betaine-based DESs function as both reaction media and structural building blocks. These solvents can facilitate organic synthesis and nanomaterial preparation. For cellulose modification, betaine-derived solvents activate biopolymers by disrupting intermolecular hydrogen bonds without altering intrinsic crystallinity, offering a pathway for derivatization [84]. This facilitates applications in biofuels and bioplastics. Specifically, pretreatment with a betaine-oxalic acid DES introduces the quaternary ammonium group into the cellulose structure, enabling the one-step preparation of zwitterionic cellulose nanofibers (Z-CNFs) [85]. The resulting Z-CNFs maintained thermal stability (Ton ≈ 190 °C), and the films exhibited high optical haze (>87%) and substantial tensile strength (96 MPa). Research also utilizes the dual functionality of betaine-xylitol DES to develop PVA-based hybrid eutectogels. In this system, the solvent served as a medium for synthesizing gold nanoparticles (AuNPs) and was subsequently integrated into the polymer network. The resulting composite displayed improved mechanical strength and pH-responsive swelling behavior [86].

In conclusion, betaine-based DESs in materials science act as “solvent-reactant-functional additives.” They demonstrate utility in modifying biological macromolecules and modulating nanocomposite performance. However, incorporating DESs into final material structures introduces challenges regarding long-term stability. The hygroscopic nature of betaine may lead to uncontrolled water absorption, potentially altering the mechanical properties of the composite material over time. Future work must address the trade-off between biodegradability and material durability under humid conditions.

## 5. Challenges and Future Directions

Despite substantial research progress, translation to clinical and industrial practice remains limited. Evidence in biomedicine and nutrition is still dominated by short-term, high-dose animal studies, which poorly represent realistic human exposure. This discrepancy raises uncertainty regarding clinical relevance and underscores the need for long-term, low-dose investigations in human populations.

Industrial application presents a different but equally restrictive set of constraints. When betaine is used as a surfactant precursor, performance is strongly influenced by feedstock purity and synthesis parameters. Zwitterionic systems show pronounced sensitivity to ionic strength and temperature, creating challenges for process stability during scale-up. Comparable limitations are observed in emerging betaine-based materials, such as deep eutectic solvents and cocrystals, where high viscosity and hygroscopicity restrict mass transfer and reduce long-term stability.

Commercialization is further constrained by economic and regulatory considerations. The production of high-purity betaine and structurally defined derivatives remains more costly than conventional petrochemical alternatives. Regulatory pathways for novel betaine-based materials, particularly nanostructured systems, are still developing and require comprehensive safety evaluation. Progress in this area will depend on improved predictive approaches, including molecular dynamics and machine-learning-based structure–property analysis, together with more economical bio-based production routes and standardized quality control frameworks.

## 6. Conclusions

This review has underscored the evolution of betaine from a classical osmoprotectant and nutritional additive to a multifunctional molecular platform driving innovations in supramolecular chemistry, green solvents, and advanced materials. As a natural zwitterion, betaine effectively bridges the gap between biological functionality and industrial utility. Its unique physicochemical structure—characterized by a permanent cationic core and a hydrogen-bond-accepting carboxylate moiety—provides a versatile foundation for constructing complex systems. These range from stress-resilient agricultural formulations and hepatoprotective therapeutic agents to cutting-edge DESs and functional cocrystals.

The synthesis of current research trends and bibliometric analysis confirms a significant transition: betaine is no longer viewed merely as a nutrient supplement but as an active, versatile building block for green molecular engineering. The emergence of betaine-based supramolecular systems has successfully addressed long-standing challenges in solubility, stability, and delivery efficiency across pharmaceutical and cosmetic sectors. Looking forward, as impediments regarding stability, predictability, and industrial scale-up are resolved through the convergence of computational modeling and experimental validation, betaine is poised to solidify its utility in sustainable chemistry. It serves as a valuable component for the development of next-generation sustainable materials, offering robust solutions that balance performance, safety, and environmental stewardship.

## Figures and Tables

**Table 2:** Betaine characteristics in biomedicine and health field and related research summary.

Function Category	Mechanism	Model/Example	Dose & Method	Key Outcome	Ref.
Alleviating Liver Injury	Restoration of SAM & GSH; enhanced antioxidant defense	Alcoholic liver injury (rat)	1% *w*/*v* betaine in liquid ethanol diet	Liver injury markers ↓	[11]
	Modulation of gut-liver axis & lipid metabolism	Mice offspring (Maternal High-Fat Diet induced NAFLD)	1% betaine supplementation to dams during pregnancy & lactation	Hepatic steatosis & Triglycerides ↓; Lipid oxidation genes (PPARα, CPT1α) ↑, TNFα ↓; Beneficial bacteria (Bacteroides) ↑ & Fecal SCFAS ↑	[12]
	Autophagy–AMPK activation; sulfur amino acid metabolism regulation	NAFLD mouse (CDAHFD model)	1% *w*/*v* betaine in water or feed	Hepatic steatosis ↓; ER stress & apoptosis ↓	[13]
	Regulation of insulin sensitivity, glucose uptake, glycogen metabolism; modulation of Notch signaling and cytochrome P450-related pathways	High-fat diet-induced hyperglycemic mice; HepG2 and C2C12 cells	1% *w*/*v* in drinking water (mice); 20 mM (in vitro cells)	Reduced blood glucose and liver triglycerides; improved glucose metabolism in liver and muscle	[14]
	Epigenetic regulation (m^6^A) & Autophagy activation	Hepatocellular carcinoma (HCC): Patients, Mice (Xenografts/Metastasis), & Cell lines	Cohort study (serum analysis); 3% *w*/*v* in drinking water (mice)	Suppressed HCC stemness & metastasis; Activated autophagy via SAM/m^6^A/YTHDF1/ATG3 axis	[15]
	Mitigation of oxidative stress & Mitochondrial protection	Animal models (Acute: TAA-induced; Chronic: Bile duct ligation)	10 & 50 mg/kg (i.p.)	Ameliorated hepatic injury & histopathology; Decreased oxidative stress markers; Preserved mitochondrial function	[16]
Preventing Cardiovascular Disease	BHMT-mediated remethylation	Hyperhomocysteinemia (human/animal)	1–2 g betaine/day, PO	Plasma tHcy significantly ↓	[17]
	Methyl donor → enhances homocysteine remethylation	Healthy adults (meta-analysis, 5 RCTs)	≥4 g/day oral betaine for ≥6 weeks	Plasma homocysteine ↓ 1.23 μmol/L (mean)	[18]
	Methyl donor activity → homocysteine remethylation	Chinese adults with hyperhomocysteinemia	Daily oral: 1 g betaine + B vitamins for 12 weeks	Plasma homocysteine ↓ 3.87 μmol/L (−10.1% vs. placebo)	[19]
	Methyl donor activity → homocysteine remethylation	Healthy, physically active males	2.5–5.0 g/day oral betaine for 21 days	Plasma homocysteine ↓ 1.5 μmol/L; no effect on lipids or enzymes	[20]
	Inhibits TNF-α and NF-κB; reduces lipid peroxidation (TBARS); boosts antioxidant enzymes (CAT, SOD, GPx); restores redox balance	Male mice with NaAsO_2_-induced cardiotoxicity	NaAsO_2_ (50 ppm, 8 weeks); BET (500 mg/kg, last 2 weeks)	Reduced cardiac oxidative damage, inflammation, enzyme leakage, and histopathological injury	[21]
Regulating Systemic Metabolism	Modulates gut microbiota and SCFA levels; activates miR-378a/YY1 pathway	HFD-fed mice; germ-free mice	1% *w*/*v* in drinking water (23 weeks for HFD mice; 45 days for Abx-treated mice)	↓ Obesity and metabolic syndrome; ↑ glucose tolerance and brown fat activity	[22]
	↑ Milk betaine → ↑ Akkermansia & goblet cells; improved gut–metabolic axis	Lactating mice & human infant cohorts	1% *w*/*v* in drinking water (supplemented to dams during lactation)	↓ Offspring adiposity; ↑ glucose homeostasis; replicated in human milk–microbiome link	[23]
	Higher plasma betaine associated with improved glucose regulation	Pregnant women with dichorionic twin gestation	Cohort study (187 twin pregnancies); Plasma analysis (median 16.1 weeks gestation)	↑ Plasma betaine linked to ↓ GDM risk (RR = 0.41, highest vs. lowest tertile)	[24]
	↓ Betaine and ↓ betaine/choline ratio linked to ↓ glucose tolerance	Pregnant women (GDM cases vs. matched controls)	Case-control study (200 cases vs. 200 controls); Serum analysis (at 24–28 weeks gestation)	Lower serum betaine associated with ↑ GDM risk and glucose excursion markers	[25]
	Regulation of steroidogenesis genes (3β-HSD, StAR, P450scc, LHR), attenuation of ER stress (GRP78, CHOP, ATF6, IRE1), activation of Nrf2 antioxidant pathways	Mouse Leydig cells	5 mM betaine, 24 h, in vitro	Enhanced cell viability and testosterone production under hyperglycemia	[26]
	Regulation of insulin sensitivity, glucose uptake, glycogen metabolism; modulation of Notch signaling and cytochrome P450-related pathways	High-fat diet-induced hyperglycemic mice; HepG2 and C2C12 cells	1% *w*/*v* in drinking water (mice); 20 mM (in vitro cells)	Reduced blood glucose and liver triglycerides; improved glucose metabolism in liver and muscle	[14]
	Enhances insulin signaling via IRS1 and Akt phosphorylation; restores hepatic betaine content; increases glycogen storage; reduces liver injury	C57BL/6J mice (high-fat diet model); primary human hepatocytes	Dietary betaine (14 weeks in vivo; 4 weeks in late-stage model); betaine treatment in vitro	Improved insulin sensitivity and hepatic insulin signaling; reduced hepatic steatosis and ALT levels	[27]
Ameliorating Muscle Atrophy	Maintains protein synthesis; upregulates Myh1 expression; counteracts TNF-α-induced morphological atrophy	C2C12 myotubes treated with TNF-α	10 mM betaine (in vitro cells treated for 72 h)	Prevented inflammatory cytokine-induced muscle atrophy in vitro	[28]
	Activates mTORC1 signaling via increased SAM levels; disrupts Samtor–mTORC1 binding; enhances myogenic factor expression and myosin heavy chain levels	Aged C57BL/6J mice; C2C12 cells	2% *w*/*v* betaine in drinking water (in vivo); betaine treatment in vitro	Improved muscle mass, strength, motor function; delayed age-related muscle atrophy	[29]
	Enhances autophagy via Mettl21c/p97/VCP axis; increases SAM levels; promotes trimethylation of p97 and autophagic flux	Aged C57BL/6J mice; C2C12 cells under methionine starvation	2% *w*/*v* betaine in drinking water (12 weeks); 10 mM betaine in vitro	Preserved autophagy markers; improved muscle mass, strength, ATP production, and cell differentiation	[30]
Anti-inflammatory and Immunomodulatory Properties	Inhibition of TLR4/NF-κB & NLRP3 inflammasome	Neuroinflammation (mouse)	In vivo administration (per study protocol)	Microglial M1 → M2 shift; pro-inflammatory cytokines ↓	[31]
	Anti-inflammatory regulation (NLRP3 inflammasome) & Homocysteine metabolism	Mice offspring (Maternal Fatty Liver Disease model: HFD + STZ)	1% betaine (Maternal supplementation during gestation & lactation)	Reversed hepatic steatosis & serum inflammation (ALT, IL-6, TNF-α); Inhibited NLRP3 inflammasome pathway (NLRP3, ASC, Caspase-1, IL-1β, IL-18); Decreased hepatic Hcy & SAH levels	[32]
	Reduces plasma SAH; increases SAM/SAH ratio; inhibits NF-κB inflammatory signaling; suppresses smooth muscle cell proliferation and migration	ApoE−/−/SAHH+/− mice fed AIN-93G diet ± betaine	4% betaine in diet for 8 weeks	Reduced atherosclerotic lesion development and vascular inflammation	[33]
	Increases antioxidant enzyme SOD; downregulates pro-inflammatory molecules (VCAM-1, IL-1β)	Lewis rats with IRBP-induced EAU	Oral betaine 100 mg/kg for 9 days	Reduced retinal and ciliary body inflammation	[34]
	Inhibits NLRP3 inflammasome and NF-κB activation, lowering inflammatory cytokines	BV2 microglial cells stimulated with Aβ(42) oligomers	2 mM betaine treatment in vitro	Reduced IL-1β, IL-18, TNF-α levels; suppressed microglial inflammation	[35]
	Inhibits NLRP3 inflammasome; shifts microglia from M1 to M2 polarization; reduces pro-inflammatory cytokines	LPS-induced depression-like behavior in ICR mice	Betaine (1% & 5% in drinking water, 21 days) + LPS injection	Improved depressive behaviors; reduced neuroinflammation	[36]
Other biological activities	Improves anabolic profile (↑ testosterone/cortisol ratio); reduces systemic inflammation	Young professional soccer players (*n* = 29, 14–15 y/o)	2 g/day oral betaine for 14 weeks	Increased VO_2_max, 1-RM strength, sprint performance, and anaerobic power	[37]
	Enhances antioxidant defense (↑ SOD, ↑ GPx, ↓ MDA); protects hippocampal neurons; reduces depressive-like behavior	Male mice exposed to ZnO nanoparticles	ZnO NPs: 600 mg/kg; Betaine: 30 mg/kg orally for 7 days	Improved FST/TST scores, restored oxidative balance, and hippocampal structure	[38]
	Inhibits microglia/astrocyte activation; shifts glial phenotypes (M1 → M2, A1 → A2); modulates cytokine profile	CFA-induced chronic pain model in rodents	600 mg/kg betaine (i.p.) daily for 14 days	Reduced pain-related depressive behavior and hippocampal neuroinflammation	[39]
	Modulates gut microbiota and SCFAs; suppresses IL-6; supports brain–gut–microbiota axis function	Mice under chronic social defeat stress (CSDS)	3% (*w*/*v*) betaine in drinking water	Prevented anhedonia-like behavior; improved microbial diversity and reduced inflammation	[40]
	Inhibits TLR4/MyD88 signaling; enhances ZO-1/occludin expression; modulates gut microbiota	ALF mouse model & IEC-18 intestinal cells	800 mg/kg/day (intragastric) for 7 days (mice); 3.4–6.8 mM (in vitro cells)	Reduced intestinal damage; improved gut barrier; restored healthy microbiota composition	[41]
	Inhibiting oxidative stress-induced pyroptosis via the ROS/NLRP3/Caspase-1/GSDMD pathway	DSS-induced ulcerative colitis (mice); IEC-18 cells (oxidative stress model)	In vivo: 20 mg/kg (oral gavage) daily; in vitro: IEC-18 cells	Reduced Disease Activity Index (DAI) & colon damage; inhibited ROS accumulation & NLRP3 inflammasome activation; decreased IL-1β & IL-18 levels	[42]
Constructing Functional Polymers	Permanent positive charge broadens the pH range of zwitterionic stability; preserves stereochemistry	Amino-acid-derived polyacrylamides (Methylated vs. Non-methylated)	Synthesis via RAFT & Post-polymerization quaternization; cell viability tested up to 1 mg mL^−1^	Superior pH stability compared to non-methylated analog; preserved chirality; high biocompatibility	[43]

Notes: ↑ indicates an increase or improvement; ↓ indicates a decrease or reduction.

**Table 4:** Betaine characteristics in food and health products field and related research summary.

Function Category	Mechanism	Model/Example	Dose & Method	Key Outcome	Ref.
Functional Food Additive	Betaine affects gas production and gluten development in dough	Bakery dough	1.5% (*w*/*w* flour basis); comparison of addition timing (initial vs. late stage)	Early addition → decreased loaf volume, increased crumb hardness; Late addition → improved loaf structure and texture	[49]
	CAPB modifies phase behavior, enhancing enzymatic selectivity	Enzymatic MAG synthesis in lipid-based formulations	5 wt% CAPB added to enzymatic reaction mixture	Improved monoacylglyceride selectivity, enhanced catalytic efficiency, formulation advantages	[50]
	Natural betaine source enriches nutrients and reduces harmful compounds	Biscuits with beetroot powder	15%, 20%, and 25% flour substitution with beetroot powder	Increased mineral and betaine content, improved sensory acceptance, reduced acrylamide levels, lower glycemic response	[51]
	Betaine safety assessment	Food additive safety testing	Up to 5000 µg/plate (Ames); up to 10 mM (TK6 cells); OECD-compliant assays	No genotoxic effects observed; validated as safe for functional food development	[52]
Food Preservation & Quality Maintenance	Alleviates chilling injury via antioxidant enzyme activation, membrane stabilization, preservation of ascorbic acid and phenolics	Cold-stored pomegranates	Exogenous betaine, 20 mM	Reduced chilling injury, enhanced antioxidant activity, maintained membrane integrity and nutritional compounds	[53]
	Modulates oxidative metabolism, membrane stabilization, sugar/energy metabolism, amino acid and protein turnover, cold-resistance gene expression	General postharvest fruits/vegetables	Review of exogenous application (typically 1–20 mM immersion)	Multifaceted mitigation of chilling injury	[54]
	Delays senescence, reduces decay, enhances antioxidant capacity and energy charge via enzyme regulation	Blueberries	Betaine treatment, 10 mM	Delayed senescence, reduced decay, improved firmness, enhanced antioxidant and energy metabolism	[55]
	Modulates ethylene production, respiration, phenylpropanoid metabolism, maintains cell wall integrity	Various postharvest fruits	Review of postharvest treatments (typically 1–20 mM dipping)	Reduced storage-related deterioration, improved postharvest physiology	[56]
	Enhances size, soluble solids, vitamin C, polyphenol content, antioxidant activity; promotes ripening	Sweet cherry, pre-harvest	Pre-harvest foliar spray (1.5 and 3.0 kg/ha)	Improved fruit quality and stress adaptation during cultivation	[57]
Flavor Modulation	Enhances feeding behavior and initiation	Aquaculture: sole	10 g/kg (1%) in feed	Improved food-seeking behavior and feeding initiation; cost remains a limitation	[58]
	Acts as flavor precursor forming volatile compounds	Plant-based seafood analogues	Added as taste-active component (approx. 0.1–0.6% *w*/*w*)	Enhanced crustacean-like aroma and consumer sensory perception via trimethylamine and pyrazines	[59]
	Synergistic enhancement of feed palatability with amino acids	Juvenile grouper	0.5%, 1.0%, 1.5%, and 2.0% in feed	Significantly increased feed preference	[60]
	Improves growth, carcass traits, and post-slaughter flavor	Ruminants	2 and 4 g/day/lamb (Rumen-protected betaine)	Enhanced meat quality: increased flavor amino acids, altered fatty acid profiles	[61]

Notes: → indicates leads to or results in.

**Table 5:** Global betaine regulations and policies.

Country/Organization	Relevant Regulations
United States	The U.S. Environmental Protection Agency (EPA) issued a notice on 7 February 2019, exempting the residue limit of betaine (CAS Reg. No. 107-43-7) when used as a plant nutrient component in pesticide formulations for crops.
European Union	In 2017, the European Food Safety Authority (EFSA) conducted a safety assessment and concluded that betaine, as a new food ingredient from sugar beet, is safe at an intake level of 400 mg/day. In 2019, the EU issued Regulation (EU) 2019/1294, approving betaine as a novel food ingredient and permitting its use in nutritional products [62].
Japan	In 1959, Japan included betaine in the “Standards for Food Additives” and established testing methods. In 1996, betaine was approved as a food additive for general consumption.
China	The 2023 edition of the “Dietary Reference Intakes for Chinese Residents” recommends a dietary intake of betaine at 1.5 g/day, with a maximum tolerable intake of 4.0 g/day.

**Table 6:** (EU) 2019/1294 Maximum use limits for betaine in specified food categories.

Food Category	Maximum Use Limits
Sports drinks, isotonic beverages, and energy drinks	60 mg/100 g
Protein and cereal bars for athletes	500 mg/100 g
Meal replacement products for athletes	20 mg/100 g
Meal replacement products for weight control as defined in Regulation (EU) No 609/2013 [63]	500 mg/100 g (solid foods) 136 mg/100 g (soups) 188 mg/100 g (porridges) 60 mg/100 g (beverages)
Foods for special medical purposes for adults as defined in Regulation (EU) No 609/2013 [63]	400 mg/day

## Data Availability

The data presented in this study are available from Clarivate Analytics at https://www.webofscience.com (accessed on 20 October 2025). These data were derived from the following resources: Web of Science Core Collection.

## Abbreviations

The following abbreviations are used in this manuscript:

AFB_1_	Aflatoxin B_1_
Akt	Protein kinase B
AMPK	AMP-activated protein kinase
APIs	Active pharmaceutical ingredients
ApoE	Apolipoprotein E
ASC	Apoptosis-associated speck-like protein containing a CARD
ATG3	Autophagy related 3
AuNPs	Gold nanoparticles
BHMT	Betaine-homocysteine methyltransferase
BLG	β-lactoglobulin
CAPB	Cocamidopropyl betaine
CI	Chilling injury
COX-2	Cyclooxygenase-2
DES	Deep eutectic solvent
EAU	Experimental autoimmune uveitis
EFSA	European Food Safety Authority
ER	Endoplasmic reticulum
GDM	Gestational diabetes mellitus
GPx	Glutathione peroxidase
HBA	Hydrogen bond acceptor
HBD	Hydrogen bond donor
HCY	Homocysteine
HFD	High-fat diet
IL-18	Interleukin-18
IL-1β	Interleukin-1β
IL-6	Interleukin-6
iNOS	Inducible nitric oxide synthase
IRS1	Insulin receptor substrate-1
LPS	Lipopolysaccharide
m6A	N6-Methyladenosine
MAG	Monoacylglycerol
MD	Molecular dynamics
Mettl21c	Methyltransferase 21C
MOS	Margin of Safety
MTG	Microbial transglutaminase
MyD88	Myeloid differentiation primary response 88
Myh1	Myosin heavy chain 1
NADES	Natural deep eutectic solvents
NAFLD	Non-alcoholic fatty liver disease
NF-κB	Nuclear factor kappa-B
NLRP3	NLR family pyrin domain containing 3
Nrf2	Nuclear factor erythroid-2 related factor 2
OECD	Organization for Economic Co-operation and Development
PBs	Polybetaines
PVA	Polyvinyl alcohol
SAM	S-adenosylmethionine
SAH	S-adenosylhomocysteine
SASA	Solvent-accessible surface area
SCFA	Short-chain fatty acid
SCG	Sodium cocoyl glycinate
SOD	Superoxide dismutase
T2DM	Type 2 diabetes mellitus
tHCY	Total homocysteine
TLR4	Toll-like receptor 4
TNF-α	Tumor necrosis factor-α
VCAM-1	Vascular cell adhesion molecule 1
VCP	Valosin-containing protein
VOC	Volatile organic compound
YTHDF1	YTH domain-containing family protein 1
Z-CNFs	Zwitterionic cellulose nanofibers
